# Is preterm birth associated with asthma among children from birth to 17 years old? -A study based on 2011-2012 US National Survey of Children’s Health

**DOI:** 10.1186/s13052-018-0583-9

**Published:** 2018-12-22

**Authors:** Jie Zhang, Chenchao Ma, Aimin Yang, Rongqiang Zhang, Jiannan Gong, Fengfeng Mo

**Affiliations:** 1 Department of Ship Hygiene, Faculty of Naval Medicine, Second Military Medical University, Shanghai, 200433 China; 2 School of Public Health, Brown University, Providence, RI USA; 3 Department of thoracic surgery, Shanghai Pulmonary Hospital, Tongji University, Shanghai, China; 4 School of Public Health, Shaanxi University of Chinese Medicine, Xianyang, China; 5 Department of Respiratory and Critical Medicine, The Second Affiliated Hospital of Shanxi Medical University, Taiyuan, China

**Keywords:** Asthma, Preterm birth, Low birth weight, National Survey of Children’s health (NSCH), Respiratory and children, United States

## Abstract

**Background:**

Preterm birth can interrupt lung development in utero and is associated with early life factors, which adversely affects the developing respiratory system. Studies on preterm birth and asthma risk are comparatively sparse and the results are not consistent.

**Methods:**

Multivariate analyses were performed on a cross-sectional data from the National Survey of Children’s Health (NSCH) collected in 2011 to 2012. The NSCH was a nationally representative telephone survey sponsored by the Maternal and Child Health Bureau and conducted by the National Center for Health Statistics. A cross-sectional analysis using data from the US on 90,721 children was conducted to examine the relationship between preterm birth and asthma risk.

**Results:**

A total of 90,721 children under 17 years were included and 12% of the children were reported as preterm birth. The prevalence of diagnosed asthma was 15%, with a male to female ratio of 1.26:1. Children who were born preterm were 1.64 times (95% confidence interval: 1.45–1.84) more likely to develop asthma compared with those who were born term after controlling for confounders. Similarly, children who were low birth weight were 1.43 times (95% confidence interval: 1.25–1.63) more likely for asthma, and the odds ratio increased to 1.77 for those both preborn and low birth weight. Child’s gender, race/ethnicity, age, family structure, family income levels, and household smoking were significantly associated with the odds of reported asthma.

**Conclusions:**

Preterm birth was associated with increased risk of asthma among US children, supporting the notion that preterm birth may play a critical role in asthma development.

**Electronic supplementary material:**

The online version of this article (10.1186/s13052-018-0583-9) contains supplementary material, which is available to authorized users.

## Background

Asthma is a heterogeneous disease, characterized by chronic airway inflammation [1], which is one of the leading chronic childhood diseases [2] and a major cause of childhood disability [3] Data from the International Study of Asthma and Allergies in Childhood (ISAAC) suggest that the prevalence of asthma symptoms became globally increased ranging form 11.1 to 11.6% in children and from 13.2 to 13.7% in adolescents from Phase one to Phase Three [4]. The prevalence rate varies among countries, the increase pattern usually observed in many Low- and Middle-Income counties, especially in Eastern Europe and Latin American, as well as Northern African countries [4, 5]. The cause of asthma remains unclear and current research paints a complex picture [6, 7].

Preterm births are one of the leading health burdens of children, which are reported to be related to increased risks of many health problems [8]. The frequency of preterm births is about 12–13% in the USA and 5–9% in many other developed countries [9]. During the past decades, secular trends of increased preterm birth and asthma prevalence in adults and children have led to a debate about potential links between the two conditions [10]. A possible mechanism to explain this association is that preterm birth is related to a deficit in the structure and function of the lung, which may increase the risk of subsequent asthma development [11]. A prospective cohort study showed that a doctor’s diagnosis of asthma and using of asthma inhalers were significantly more prevalent among preterm birth children than controls [12]. Another retrospective cohort study also showed an important association between later preterm birth (between 34 weeks and 36 weeks) and incidences of asthma [13]. Effect estimates from a meta-analysis of studies on the association between preterm birth and the risk of asthma showed that infants born preterm (defined as < 37 weeks of gestation) could have up to 36% greater risk of asthma than infants born at term [14].

Some researchers found no correlation between preterm birth and a doctor’s diagnosis of asthma [15], and argued the association might be due to residual non-genetic confounding factors [16]. However, most of these studies were hospital-based instead of population-based, the possibility of selection bias can’t be ruled out. In addition, there were limited study sample size and different ages of study population. In order to have a better understanding whether preterm birth plays a role in the development of asthma, we used the data from the 2011–2012 National Survey of Children’s Health (NSCH), which contains 95,677 samples that are geographically and economically diverse to assess the association between preterm birth and asthma. We also explored the possible combined effect of children’s age, gender, race, household smoking, neighborhood garbage exposure, family structure, income levels and parents’ education level.

## Methods

### Study population

**Fig. 1:**
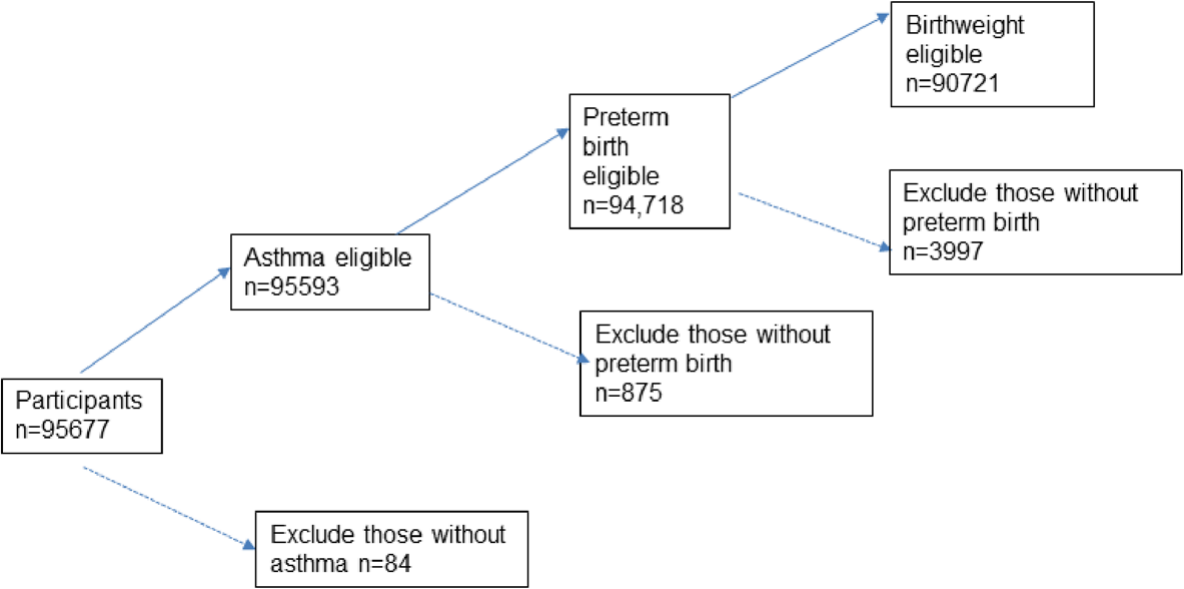
Study population. Note: Dash lines indicate when participants were excluded from the study; Solid lines indicate when participants were included in the study

This was a secondary analysis using the third survey data from the NSCH, which was conducted between 2011 and 2012. The NSCH was a cross-sectional, nationally representative telephone survey sponsored by the Maternal and Child Health Bureau and conducted by the National Center for Health Statistics [17]. The study population consisted of children aged 0–17 in the United States. Eligible participants in NSCH were 95,677. The analytic sample should contain valid information of the outcome including asthma and primary predictor (preterm birth and birth weight), respondents with don’t know/refuse/missing were excluded. Thus, a sample of 90,721 (90%) children under 17 year’s old constituted present study (See Fig. 1).

### Outcome

The outcome of interest was asthma. According to World Health Organization (WHO) guideline, asthma is a chronic disease characterized by recurrent attacks of breathlessness and wheezing [18]. In this study, asthma was measured using respondent (primary caregiver) reports in the NSCH survey of whether “a doctor or health professional ever told you that the child has asthma”.

### Predictor variable

The primary predictor variable was preterm birth, which means the birth of an infant before 37 weeks of pregnancy [19]. In this study, children classified as born preterm were those with a parent report yes for the question “Was [S.C.] born prematurely, that is, more than 3 weeks before [his/her] due date?” The secondary predictor was low birth weight as some cross-sectional studies showed the impact of low birth weight on early-childhood asthma [20]. According to WHO guideline, infants with a birth weight of less than 2500 g, are considered as low birth weight [21]. In this study, respondents provided answers for the question “what was [SC] birth weight”, we divided the parent-reported birth weight into three categories: 1. > =2500 g, 2. 2000~2499 g, 3. < 2000 g, category 2 and 3 were defined as the low birth weight.

### Confounding variables

Covariates to control for potential confounding were chosen on the basis of asthma risk factors in previous studies. The confounders included children age, gender, race, family member education level, family structure and income level, household smoking, and neighborhood garbage exposure. Although other factors such as breastfed might have been useful to evaluate, there was a skip pattern in the survey and the data was not reliable for analyzing this factor.

Demographic variables including children age and gender were provided by respondents in the NSCH survey. Race or ethnicity was categorized as Hispanic, non-Hispanic-white, non-Hispanic-black or other; Family member education level was defined as highest level of education obtained by anyone in household (< high school, high school degree, > high school). Family structure was categorized as currently married, cohabiting, living apart, never married, or no parents in household. Family income levels were derived from the question Since [S.C.] was born, how often has it been hard to get by on your family’s income, for example, it was hard to cover the basics like food or housing?], the responses were coded as never, not very often, somewhat often, and very often. It was recently reported that children asthma was associated with indoor and outdoor pollution [22, 23], thus household smoking and neighborhood garbage exposure were considered in our study. Household smoking was defined as a dichotomous variable indicating whether “anyone in the household uses cigarettes, cigars, or pipe tobacco”. Neighborhood garbage was defined as response to “In your neighborhood, is there litter or garbage on the street or sidewalk?” (Additional file 1: Table S1).

### Statistical analysis

First, correlations between variables were checked. The multicollinearity between variables was explored, and the results indicated that multicollinearity was not a big problem (Additional file 1: Table S1). Next, multivariate logistic regression analysis was used to analyze the association between preterm birth and the binary outcome asthma, with the adjustment for all other confounders, including children age, gender, race, family education level, family structure, family income levels, household smoking and garbage exposure. Based on Directed acyclic graphs (DAGs), low birth weight might be on the causal pathway between preterm birth and asthma, therefore it would be inappropriate for inclusion in the same logistic regression model with preterm birth. Thus, three separate models were conducted. Model1 and model2 investigated the individual effect of preterm birth and low birth weight separately adjusting for other confounders. Model 3 investigated the combined effect of preterm birth and low birth weight. Last but not least, interaction terms of gender and race were added to the base model1 and model2. Because no interaction terms remained statistically significant after the adjustment of the significance level for multiple testing, the final model did not include any interaction terms. Models were estimated after taking account of weighting and complex survey design. A *p*-value of < 0.05 was considered statistically significant. All analyses were performed using Stata 13.0 (Stata Corp, College Station, TX).

### Ethics

All research was approved by institutional review boards (IRB). Respondents were informed that the study was voluntary and confidential at the beginning of the survey. Verbal consent for participation in the study was obtained from NSCH respondents as the survey was conducted via telephone.

## Results

Based on the parent-report, the overall prevalence of preterm birth was 12% and that of asthma was 15%, with a male to female ratio of 1.26:1. The gender distribution of the study population was relatively equal. 51% of the children in the study were non-Hispanic white, followed by Hispanic (21%), non-Hispanic black (17%), and other non-Hispanic (11%) among children born preterm. There was a similar distribution among those who were born at term. The majority of children (47%) were in households where at least one resident had more than a high school education. Children who were preborn tend to live with parents not currently married (62% versus 62%), who lived apart (15% versus13%), never married (11% versus 8.8%) or no parents in household (2.7% versus 2.4%) compared to those who were term born. What’s more, they come from family who were more likely to report very often difficult to pay for bills (8.7% compared to 6.7%). 27% of children who were born preterm lived in households with someone who used of cigarettes, cigars, or pipe tobacco, while 24% of those were born at term; 16% of children lived in a neighborhood with litter or garbage on the street or sidewalk, there was no difference between preterm- and term-born children (Table 1). We also compared the distribution of the above covariates by low birth weight, which indicated similar trend with preterm birth.

**Table 1:** Characteristics of U.S. Children between birth and 17 years old by birth characteristics, NSCH (2011–2012)

Variable	Total	Term born(*n* = 80,295)	Preterm birth(*n* = 10,426)	*P value*	Normal Birth Weight(*n* = 82,519)	Low Birth Weight(*n* = 8202)	*P value*
	Unweighted n(weight%)	Unweighted n(weight%)	Unweighted n(weight%)		Unweighted n(weight%)	Unweighted n(weight%)	
Asthma				< 0.001*			< 0.001*
Yes	12,839 (15%)	10,763 (14%)	2076 (21%)		11,294 (14%)	1545 (19%)	
No	77,882 (85%)	69,532 (86%)	8350 (79%)		71,225 (86%)	6657 (81%)	
Gender of Child				0.337			< 0.001*
Male	46,673 (51%)	41,091 (51%)	5582 (52%)		42,763 (52%)	3910 (46%)	
Female	43,953 (49%)	39,115 (49%)	4838 (48%)		39,671 (48%)	4282 (54%)	
Child’s age at interview (years)				0.0075*			0.0307
<=3	19,036 (22%)	16,848 (22%)	2188 (23%)		17,379 (22%)	1657 (22%)	
4~6	14,912 (17%)	13,031 (17%)	1881 (19%)		13,383 (17%)	1529 (20%)	
7~12	29,915 (33%)	26,351 (33%)	3564 (33%)		27,077 (33%)	2838 (33%)	
13~17	26,858 (28%)	24,065 (28%)	2793 (58%)		24,680 (28%)	2178 (26%)	
Race/Ethnicity of Child				0.001*			< 0.001*
Hispanic	11,758 (23%)	10,417 (23%)	1341 (21%)		10,525 (23%)	1233 (24%)	
Non-Hispanic white	59,186 (54%)	52,727 (54%)	6459 (51%)		54,690 (55%)	4496 (42%)	
Non-Hispanic black	8231 (13%)	7069 (13%)	1162 (17%)		6977 (12%)	1254 (23%)	
Non-Hispanic other	9472 (10%)	8271 (10%)	1201 (11%)		8474 (10%)	998 (11%)	
Parents’ Education				0.89			0.0149
Less than high school	11,903 (20%)	10,459 (20%)	1444 (20%)		10,569 (20%)	1334 (22%)	
High school graduate	29,176 (32%)	25,784 (32%)	3392 (33%)		26,539 (32%)	2637 (33%)	
More than high school	44,745 (48%)	39,774 (48%)	4971 (47%)		41,042 (48%)	3703 (45%)	
Family Structure							
Currently married	64,235 (66%)	57,302 (67%)	6933 (62%)	< 0.001*	59,112 (67%)	5123 (58%)	< 0.001*
Cohabiting	5533 (8.8%)	4839 (8.7%)	694 (9.7%)		4909 (8.7%)	624 (10%)	
Living apart	10,559 (14%)	9153 (13%)	1406 (15%)		9459 (13%)	1100 (16%)	
Never married	6674 (9.1%)	5764 (8.8%)	910 (11%)		5843 (8.8%)	831 (12%)	
No parents in household	2678 (2.4%)	2321 (2.4%)	357 (2.7%)		2263 (2.3%)	415 (3.8%)	
Difficulty paying for bills				< 0.001*			
Never	43,447 (43%)	38,913 (43%)	4534 (39%)		39,832 (43%)	3615 (42%)	
Not very often	26,836 (32%)	23,745 (32%)	3091 (31%)		24,510 (32%)	2326 (30%)	
Somewhat often	13,920 (19%)	12,075 (18%)	1845 (21%)		12,415 (19%)	1505 (20%)	
Very often	4927 (6.9%)	4144 (6.7%)	783 (8.7%)		4334 (6.8%)	593 (8.3%)	
Use of cigarettes, cigars, or pipe tobacco in the household				0.0013*			0.0094*
Yes	20,950 (24%)	18,254 (24%)	2696 (27%)		18,714 (24%)	2236 (27%)	
No	68,942 (76%)	61,303 (76%)	7639 (73%)		63,061 (76%)	5881 (73%)	
Litter or garbage on the street or sidewalk				0.7236			0.3326
Yes	13,341 (16%)	11,772 (16%)	1569 (16%)		12,058 (16%)	1283 (17%)	
No	76,024 (84%)	67,321 (84%)	8703 (84%)		69,241 (84%)	6783 (83%)	

*p < 0.05

Univariate logistic regression model for the potential predictors and asthma were performed and the results are displayed in Table 2. In the unadjusted model, children who were preterm birth were 1.67 times (95% CI: 1.49–1.86) more likely to develop asthma compared with those who were born at term. The odds ratio of low birth weight for asthma was 1.46 (95%CI: 1.29–1.65) (Table 2).

**Table 2:** Univariate logistic regression of associated factors for Asthma, NSCH (2011–2012)

Variable	Univariate		
	OR	95% CI	
Preterm birth	1.67	1.49	1.86
Low Birth Weight	1.46	1.29	1.65
Gender of Child (Male)	1.26	1.16	1.37
Race/Ethnicity of Child			
Hispanic	Ref		
Non-Hispanic white	0.99	0.88	1.12
Non-Hispanic black	1.94	1.67	2.24
Non-Hispanic other	1.15	0.97	1.35
Child’s age at interview (years)			
< =3	Ref		
4~6	2.36	2.01	2.78
7~12	2.89	2.51	3.32
13~17	3.51	3.04	4.05
Family Structure			
Currently married	Ref		
Cohabiting	1.08	0.92	1.26
Living apart	1.47	1.31	1.66
Never married	1.71	1.49	1.95
No parents in household	2.20	1.77	2.72
Difficulty paying for bills			
Never	Ref		
Not very often	1.25	1.13	1.38
Somewhat often	1.45	1.30	1.62
Very often	1.95	1.67	2.27
Parents’ Education			
More than high school	Ref		
High school graduate	1.10	1.01	1.21
Less than high school	1.13	1.00	1.28
Use of cigarettes, cigars, or pipe tobacco in the household	1.39	1.27	1.52
Litter or garbage on the street or sidewalk	1.20	1.08	1.34

*Ref* Reference

*p < 0.05

Three multivariate logistic models were conducted to adjust for other covariates. Model1 and model2 investigated the individual effect of preterm birth and low birth weight separately adjusting for other confounders. Model 3 investigated the combined effect of preterm birth and low birth weight. Odds ratios of reporting asthma among preterm-born children were 1.64 (95% CI: 1.45–1.84) after adjusting for child gender, age, race, parents’ education level, family structure, family income levels, household smoking and neighborhood garbage status (Table 3). Low birth weight was also associated with asthma, with the odds ratio of 1.43 (95%CI: 1.25–1.63%). When considering the combined effect of preterm birth and low birth weight, the odds ratios increased to 1.77 (95%CI: 1.52–2.07).

**Table 3:** Multivariate logistic regression of birth characteristics for Asthma, NSCH (2011–2012)

Variable	Multivariate Logistic Regression								
	Model 1^a^			Model 2^b^			Model 3^c^		
	OR	95% CI		OR	95% CI		OR	95% CI	
Preterm birth	1.64*	1.45	1.84	NA			NA		
Low birth weight				1.43*	1.25	1.63	NA		
Preterm birth and Low birth weight							1.77*	1.52	2.07
Gender of Child (Male)	1.29*	1.19	1.41	1.31*	1.20	1.43	1.30*	1.20	1.42
Race/Ethnicity of Child									
Hispanic	Ref			Ref			Ref		
Non-Hispanic white	0.94	0.81	1.08	0.95	0.82	1.10	0.94	0.82	1.09
Non-Hispanic black	1.61*	1.36	1.90	1.60*	1.36	1.89	1.59*	1.35	1.88
Non-Hispanic other	1.14	0.95	1.36	1.15	0.96	1.38	1.15	0.96	1.37
Child’s age at interview (years)									
< =3	Ref			Ref			Ref		
4~6	2.44*	2.06	2.89	2.44*	2.06	2.89	2.44*	2.06	2.89
7~12	3.12*	2.69	3.60	3.11*	2.68	3.60	3.12*	2.70	3.61
13~17	3.84*	3.30	4.46	3.80*	3.27	4.42	3.83*	3.29	4.46
Family Structure									
Currently married	Ref			Ref			Ref		
Cohabiting	1.17	0.98	1.39	1.17	0.98	1.39	1.17	0.98	1.39
Living apart	1.16*	1.02	1.32	1.16*	1.02	1.32	1.16*	1.02	1.31
Never married	1.40*	1.20	1.64	1.40*	1.20	1.64	1.40*	1.20	1.64
No parents in household	1.61*	1.27	2.04	1.59*	1.25	2.02	1.60*	1.26	2.02
Difficulty paying for bills									
Never	Ref			Ref			Ref		
Not very often	1.13*	1.02	1.26	1.14*	1.03	1.26	1.14*	1.02	1.26
Somewhat often	1.29*	1.14	1.46	1.30*	1.15	1.47	1.30*	1.15	1.47
Very often	1.61*	1.35	1.92	1.64*	1.38	1.95	1.62*	1.36	1.93
Parents’ Education									
More than high school	Ref						Ref		
High school graduate	0.97	0.88	1.07	0.97	0.88	1.06	0.97	0.88	1.07
Less than high school	0.94	0.82	1.08	0.93	0.82	1.07	0.94	0.82	1.07
Use of cigarettes, cigars, or pipe tobacco in the household	1.19*	1.08	1.31	1.20*	1.08	1.32	1.19*	1.08	1.31
Litter or garbage on the street or sidewalk	1.08	0.95	1.21	1.07	0.95	1.20	1.07	0.95	1.21

^a^ Predicting asthma with Preterm birth

^b^ Predict asthma with low birth weight

^c^ Predict asthma with both preterm birth and low birth weight

Ref, Reference

*p < 0.05

Child’s gender, race/ethnicity, age, family structure, family income levels, and household smoking were found to be significantly associated with the odds of reported asthma in the fully adjusted model (Table 3). Male children had a substantially higher likelihood of being diagnosed with asthma compared with females (aOR = 1.29, 95% CI: 1.19–1.41). The reported asthma prevalence rate increased with age. Preterm-born children were more likely to be non-Hispanic Black (aOR = 1.61, 95% CI: 1.36–1.90) than non-Hispanic White (aOR = 0.94, 95% CI: 0.81–1.08) or other non-Hispanic (aOR = 1.14, 95% CI: 0.95–1.36). Family background also plays a critical role, children who were preborn were from families that the parents living apart (aOR = 1.16, 95%CI: 1.02–1.32), never married (aOR = 1.40, 95%CI: 1.20–1.64), or no parents in household (aOR = 1.61, 95%CI: 1.27–2.04), and families were 1.61 times more likely to feel very often difficult to pay for bills. With respect to smoking habits, results showed that having a smoker in the household increased the odds ratio of asthma by 31%. The odds ratio of litter or garbage exposure became not significant in the multivariable model. Similarly, parents’ education levels were not associated with reported asthma.

## Discussion

Our study explored the relationship between birth characteristics (preterm birth and low birth weight) and asthma among children aged 0–17 years in the 2011–2012 NSCH survey. The results demonstrated that children who were born preterm were more likely to develop asthma compared with those who were born at term. We also found low birth weight as an independent risk factor for asthma. It is noteworthy that those who were both preterm born and low birth weight had higher odds ratios for asthma. Covariates, including male sex, black race, age, parent’s not currently married, low income levels, and household smoking remained significant in the model, indicating that they were stronger predictors for developing asthma. The implication that children that preborn or low birth weight have a propensity for respiratory disease, is consistent with the results of previous studies [10, 24–26].

This study was based on the NSCH database, which is the largest, most comprehensive survey on the health of children in the United States to date. The 2011–2012 NSCH study covered 95,677 sampled children aged 0–17 years, with geographic distribution and cultural diversity. Thus findings deriving from the dataset could reflect the situation of United States generation. The analytic sample size was 90,721, which accounted for 90% of the eligible population. Sensitivity analysis showed that there was no difference in excluded observations due to missing or invalid information. The large sample size in this analysis allows a more precise estimate of the relationship between preterm birth or low birth weight and the development of asthma.

Previous studies have reported that the prevalence rates of asthma varied from 8.3 to 15% by the standard interviews or self-administered questionnaires [10, 24]. Our population-based parental-reported asthma rate was 15%, which was similar to above results. Although a number of studies have been conducted to explore the risk factors for asthma, especially the possible role of preterm birth, the results still remain inconsistent. A retrospective cohort study indicated that late-preterm gestation (34–36 completed gestational weeks) was associated with significant increases in persistent asthma diagnoses (adjusted odds ratio [aOR]: 1.68) compared with term gestation [25]. Escobar et al. [26] also reported a statistically significant association between late prematurity and recurrent wheeze in children up to 3 years of age. Abe and colleagues ^14^ used the Third National Health and Nutrition national Survey (1988–1994), found that asthma was associated with late preterm birth, but the result was not statistically significant. However, a recent cohort study conducted in US reported that late-preterm and term birth was not independently associated with a risk of asthma [27]. A recent meta-analysis covering 147,000 European children found preterm birth was positively associated with an increased risk of preschool wheezing (pOR = 1.34, 95%CI: 1.25–1.43) and school-age asthma (pOR = 1.40, 95%CI: 1.18–1.67) independent of birth weight. In line with this study, we also found the positive relationship between preterm birth and self-reported asthma. There are many factors that could explain the discrepancy in their findings. For example, the sample size for late term birth tended to be small which would limit the statistic power. The analytical approach and the adjustment for confounders also vary among studies.

In our study, covariates including children age, gender, race, parent education level, indoor and outdoor pollution exposure were controlled in the analysis. Our results complied with other studies that male children had a higher likelihood of being diagnosed with asthma compared with females [28], though the etiology remains unclear. We also found that age was a significant risk factor for asthma. In the contract, a large-scale cohort study [29] and a cross-sectional study [30] conducted in Korea failed to find that the asthma prevalence was significantly associated with age. Race disparity was found in the study. Non-Hispanic black children were substantially more likely to be reported with asthma compared with children who were non-Hispanic white or other non-Hispanic. Some researches acclaimed that gender and race might have modification effect on asthma [31]. We did the interaction effect, however, no significant results were found. This may be due to the different distribution of race around the world [32, 33].

In addition, effects of indoor and outdoor pollution exposure were also considered. Several studies had reported that smoking was significantly associated with the risk of asthma, and should always be considered in the models [34]. This analysis confirmed that smoking is a risk factor for asthma. Results showed that having a smoker in the household increased the odds ratio of asthma by 31%. Nevertheless, due to the question design, we could not test the magnitude effect of passive smoking. Neighborhood environment has also been an important consideration in asthma disparities [35]. We found difference in the distribution of living in neighborhood with litter or garbage on the street or sidewalk among children who developed asthma, however, the odds ratios were not significant in the adjusted models. Family structure and income levels are significant predictors. The children who reported asthma were more likely to from family members that not currently married and felt difficult to pay for bills, which implied unsecure living environment. Those psychosocial stressors are highly related to the onset of asthma.

This finding offers practical prevention strategy for asthma. Avoidance of being exposed to smoking, pollutants or dust in the early life stage, and social care for children from low income background would attribute to the control of asthma development.

Some limitations should be considered in the interpretation of our results. In terms of outcome measurement, we used parental report of asthma as the outcome. Parental reporting bias might exist. Parents might not recognize the problems or not consider it serious enough for consulting or diagnosis. The prevalence of asthma could be underreported because of limited access to health care services. Dependence on parental reports may underestimate the prevalence of asthma, which might have biased our results toward the null. What’s more, the NSCH measurement of asthma prevalence was based on a single question. There was no hierarchy of asthma outcomes, which limited our ability to compare the relationship between preterm birth and the extent of asthma. Similarly, preterm birth (the primary predictor) was also based on parental report on a single question that “Was [S.C.] born prematurely, that is, more than 3 weeks before [his/her] due date?” An analysis comparing clinical estimates and LMP estimates of gestational age showed that infants born at 28–36 weeks gestation were prone to show misclassification error [36]. The nature of the question also confines our ability to analyze the possible different effect between late preterm birth and early preterm birth. In general, infants born late preterm who survive the neonatal period are healthier than those who are early preterm birth. Thus, selective survival might have biased our results toward the null. What’s more, as part of the NSCH survey, we were not able to accurately assess the impact of some factors on respiratory outcomes, such as allergens in the home [36], family history of asthma [37], delivery method [38], breastfeeding [38] and atopy [38, 39]. Combining clinical records in the analysis may compensate for the disadvantage in future research. Lastly, this is a cross-sectional study, so it is impossible to establish the causal relationship of these factors. Although preterm birth happened before the diagnose of asthma, we can’t rule out the possibility that some common risk factors cause both preterm birth and can influence the onset of asthma prenatally.

## Conclusions

Despite limitations listed above, this study provides insights into the research on the relationship between preterm birth and asthma. Compared with infants born at term, infants born prematurely are more susceptible to respiratory morbidities during their birth hospitalization and neonatal period [40]. Preterm birth may affect the pathogenesis of asthma and/or contribute to asthma morbidity by triggering exacerbations through neuro-immunologic mechanisms [14]. Because asthma rates have continued to increase, contributing substantially to morbidity and health care spending for children [41], pediatric and obstetric providers, as well as families, should recognize the role that early gestation might play in the development of asthma.

In conclusion, our study demonstrated that preterm birth was a risk factor for asthma with sufficient statistic power in a nationally representative sample. Additional longitudinal researches are needed to confirm the observation and establish the causal inference relationship.

## Additional file

Additional file 1: **Table S1.** Variables, Original Survey Questions, and Recoded factors Used in analyses of 2011-2012 data from NSCH. **Table S2.** Correlations among preterm birth and other covariates. (DOCX 23 kb)

## Abbreviations

DAGs: Directed acyclic graphs
NSCH: National Survey of Children’s Health
WHO: World Health Organization
