# Supplementary material for: Is preterm birth associated with asthma among children from birth to 17 years old? -A study based on 2011-2012 US National Survey of Children’s Health
Source: Ital J Pediatr. 2018 Dec 22;44:151. doi: 10.1186/s13052-018-0583-9 (PMC6303925; doi:10.1186/s13052-018-0583-9)
Supplement: Supplementary file 1 — Table S1. Variables, Original Survey Questions, and Recoded factors Used in analyses of 2011-2012 data from NSCH. Table S2. Correlations among preterm birth and other covariates. (DOCX 23 kb) [file 13052_2018_583_MOESM1_ESM.docx]

| **Table S1. Variables, Original Survey Questions, and Recoded factors Used in analyses of 2011-2012 data from NSCH** | | |
| --- | --- | --- |
| **Variable** | **National Survey of Children’s Health Questions** | **Recoded for Descriptive Analyses** |
| Asthma | Has a doctor or other health care provider ever told you that S.C. had asthma? | Yes |
|  |  | No |
| Preterm birth | Was S.C. born prematurely, that is, more than 3 weeks before [his, her] due date? | Yes |
|  |  | No |
| Birth weight | What was S.C. birth weight in pounds? | 1. Birthweight>=2500; |
|  |  | 2. Birthweight>=2000&<2500; |
|  |  | 3. Birthweight<2000 |
| Sex | Sex of selected child | Male/ Female |
|  |  |  |
| Age | Child’s age in years at interview | Continuous |
| Race | Race classification for all states | Hispanic |
|  |  | Non-Hispanic-white |
|  |  | Non-Hispanic-black |
|  |  | Other |
| Parents Education | What is the highest grade or year of school completed | Less than high school |
|  |  | High school graduate |
|  |  | More than high school |
| Difficulty paying for bills | Since [S.C.] was born, how often has it been hard to get by on your family’s income, for example, it was hard to cover the basics like food or housing?] | Never |
|  |  | Not very often |
|  |  | Somewhat often |
|  |  | Very often |
| Cigarettes, cigars, or pipe tobacco use at the household | Does anyone live in your household use cigarettes, cigars or pipe tobacco? | Yes |
|  |  | No |
|  |  |  |
| Litter or garbage on the street or sidewalk | In your neighborhood, is there litter or garbage on the street or sidewalk? | Yes |
|  |  | No |

| **Table S2. Correlations among preterm birth and other covariates** | | | | | | | | | | | |
| --- | --- | --- | --- | --- | --- | --- | --- | --- | --- | --- | --- |
|  | Asthma | Preborn | Birthweight | Gender | Age | Race/Ethnicity | Family structure | Difficult paying bills | Education | Smoking | Garbage |
| Asthma | 1.00 |  |  |  |  |  |  |  |  |  |  |
| Preborn | 0.06 | 1.00 |  |  |  |  |  |  |  |  |  |
| Birthweight | -0.04 | -0.49 | 1.00 |  |  |  |  |  |  |  |  |
| Gender | 0.06 | 0.02 | 0.09 | 1.00 |  |  |  |  |  |  |  |
| Age | 0.13 | -0.02 | 0.05 | 0.01 | 1.00 |  |  |  |  |  |  |
| Race/Ethnicity | 0.04 | 0.02 | -0.05 | -0.01 | -0.02 | 1.00 |  |  |  |  |  |
| Family structure | 0.08 | 0.03 | -0.10 | 0.00 | -0.02 | 0.10 | 1.00 |  |  |  |  |
| Difficult paying bills | 0.08 | 0.04 | -0.05 | 0.00 | 0.02 | -0.02 | 0.22 | 1.00 |  |  |  |
| Education | 0.04 | 0.01 | -0.05 | 0.00 | 0.08 | -0.09 | 0.16 | 0.16 | 1.00 |  |  |
| Smoking | 0.04 | 0.02 | -0.05 | 0.00 | 0.00 | 0.04 | 0.15 | 0.22 | 0.11 | 1.00 |  |
| Garbage | 0.02 | 0.00 | -0.01 | -0.01 | -0.06 | 0.06 | 0.08 | 0.12 | 0.04 | 0.06 | 1 |
